# Simultaneous changes in anthocyanin, chlorophyll, and carotenoid contents produce green variegation in pink–leaved ornamental kale

**DOI:** 10.1186/s12864-021-07785-x

**Published:** 2021-06-17

**Authors:** Yang Liu, Xin Feng, Yuting Zhang, Fuhui Zhou, Pengfang Zhu

**Affiliations:** 1 College of Forestry, Shenyang Agricultural University, Shenyang, 110866 China; 2 Key Laboratory of Forest Tree Genetics, Breeding and Cultivation of Liaoning Province, Shenyang, 110866 China

**Keywords:** Anthocyanin, Carotenoids, Chlorophyll, Leaf color, Ornamental kale, Transcriptome analysis, Variegated leaf

## Abstract

**Background:**

Anthocyanin, chlorophyll, and carotenoid pigments are widely distributed in plants, producing various colors. Ornamental kale (*Brassica oleracea var. acephala* DC) which has colorful inner leaves is an ideal plant to explore how these three pigments contribute to leaf color. The molecular mechanisms of the coloration in ornamental kale could provide reference for exploring the mechanisms of pigmentation in other plants.

**Results:**

In this study, we sequenced the transcriptome and determined the pigment contents of an unusual cultivar of ornamental kale with three different types of leaf coloration: pink (C3), light pink (C2), and variegated pink–green (C1). A total of 23,965 differentially expressed genes were detected in pairwise comparisons among the three types of leaves. The results indicate that *Bo9g058630* coding dihydroflavonol 4–reductase (DFR) and *Bo3g019080* coding shikimate O–hydroxycinnamoyltransferase (HCT) acted in anthocyanin biosynthesis in pink leaves. *Bo1g053420* coding pheophorbidase (PPD) and *Bo3g012430* coding 15–cis–phytoene synthase (crtB) were identified as candidate genes for chlorophyll metabolism and carotenoid biosynthesis, respectively. The transcription factors TT8, MYBL2, GATA21, GLK2, and RR1 might participate in triggering the leaf color change in ornamental kale. Anthocyanin content was highest in C3 and lowest in C1. Chlorophyll and carotenoid contents were lowest in C2 and highest in C1.

**Conclusions:**

Based on these findings, we suspected that the decrease in anthocyanin biosynthesis and the increase in chlorophyll and carotenoid biosynthesis might be the reason for the leaf changing from pink to variegate pink–green in this unusual cultivar. Our research provides insight into the molecular mechanisms of leaf coloration in ornamental kale, contributing to a theoretical foundation for breeding new varieties.

**Supplementary Information:**

The online version contains supplementary material available at 10.1186/s12864-021-07785-x.

## Background

The wide variety of colors displayed by plant leaves, flowers, and fruits are mainly produced by combinations of three types of pigments: anthocyanins, chlorophyll, and carotenoids. Anthocyanins are water–soluble and widely present in plant vacuoles [1]. Depending on the types and amounts of anthocyanins present, plant tissues can appear purple, red, pink, or blue in color. Anthocyanins also act as antioxidants [2]. Chlorophylls are the main photosynthetic pigments in plants; the main types are chlorophyll *a*, which is blue–green, and chlorophyll *b*, which is yellow–green under light conditions [3]. Carotenoids are also photosynthetic pigments, and help to protect the photosynthetic apparatus from photo–oxidation [4]. Depending on the distribution of carotenoids, plants can appear yellow, orange, or red.

In plants, the anthocyanin biosynthetic pathway is well understood [5, 6]. Comparative transcriptome analysis of purple potato (*Solanum tuberosum*) revealed several structural genes involved in anthocyanin biosynthesis, such as those encoding phenylalanine ammonia–lyase (PAL), chalcone synthase (CHS), dihydroflavonol 4–reductase (DFR), and anthocyanin synthase (ANS) [7]. A light–induced *MYB* gene regulates anthocyanin biosynthesis in red apples (*Malus domestica* Borch) [8]. Chromatin immunoprecipitation analysis revealed that *PIF3* and *HY5* simultaneously regulate anthocyanin biosynthesis in *Arabidopsis thaliana* [9].

Chlorophyll biosynthesis and chloroplast development are well studied in rice (*Oryza sativa*) [10], *Brassica rapa* [11], *Brassica oleracea* [12], *A. thaliana* [13, 14], and other plants. Fine mapping revealed that *OsHemA* is essential for chlorophyll biosynthesis in rice [15]. Map–based cloning and sequencing of the *FLU* gene suggests that *FLU* is a negative regulator of chlorophyll biosynthesis in *A. thaliana* [16].

The carotenoid biosynthetic pathway is also well understood [17]. Carotenoids are produced by two independent pathways: the 2–C–methyl–D–erythritol–4–phosphate (MEP) pathway in plastids, and the mevalonate (MVA) pathway in the cytosol [18]. Under dark conditions, upregulation of *PSY* (phytoene synthase) leads to increased carotenoid content in *A. thaliana* [19]. *CRTISO*, encoding carotenoid isomerase, and *ε–LCY*, encoding epsilon lycopene cyclase, are two core genes related to specific orange pigmentation in *L. tulipifera* [20]. In *Brassica campestris*, *LCYE*, *LCYE2*, *CCD*, and *ZDS* were upregulated in yellow leaves relative to dark green leaves [18]. *Br–or* was identified as regulator of carotenoid biosynthesis in *B. rapa* [21]. *crtB*, encoding *Erwinia uredovora* phytoene synthase, is important in the accumulation of carotenoids in tomato fruits. Overexpression of *crtB* resulted in a significant increase in carotenoid content in tomato fruits [22].

Ornamental kale (*Brassica oleracea var. acephala* DC.), a horticultural variety, is a biennial foliage herb with leaves of various shapes and colors. Leaf color is a key commercial trait of ornamental kale [23]. At the rosette stage, the inner leaves appear purple, red, pink, light pink, white, or other colors, while the outer leaves are deep purple or green [24]. Anthocyanins, chlorophylls, and carotenoids are the most important pigments determining leaf color in ornamental kale. Cyanidin was the main type of anthocyanin in purple leaves of ornamental kale, while no anthocyanin was detected in white–leaved cultivars [25]. Fine mapping revealed that *Bo9g058680* controls red and purple leaf phenotypes in ornamental kale [26, 27]. Regulatory factors related to anthocyanin biosynthesis in *B. oleracea* have been identified using RNA sequencing (RNA–seq). MYB, bHLH, and WD40 transcription factors are essential for regulating anthocyanin biosynthesis; *R2R3–MYB*, *BoTTG1*, *BoTT8*, *BoMYBL2*, and *BoTT19* are particularly important for anthocyanin biosynthesis in ornamental kale [28]. Structural genes such as *C4H*, *TIR1*, and *LBD39*, and transcription factors such as NAC and WRKY, related to anthocyanin biosynthesis in a bicolor ornamental kale with green margins and red centers, were identified using RNA–seq [29]. A comparison of the transcriptomes of purple and white ornamental kales identified *BoC4H2*, *BoUGT9*, and *BoGST21* as candidate genes involved in the anthocyanin biosynthetic pathway, and six unigenes, *BoHEMA1*, *BoCRD1*, *BoPORC1*, *BoPORC2*, *BoCAO*, and *BoCLH1*, as related to chlorophyll metabolism [30]. RNA–seq indicated that the inhibition of chlorophyll biosynthesis probably led to the change in leaf color from green to white in ornamental kale [24].

In this study, we analyzed the molecular mechanism of leaf color change in a triple color ornamental kale cultivar. We identified differentially expressed genes (DEGs) involved in the biosynthesis of anthocyanin and carotenoid, and chlorophyll metabolism through RNA–seq. Pigment contents of the leaves of three different colors were also evaluated. Our results provide insight into the molecular mechanisms of leaf color in ornamental kale, and a reference for exploring the mechanisms of pigmentation in other plants.

## Results

### RNA sequencing and sequence assembly

**Fig. 1:**
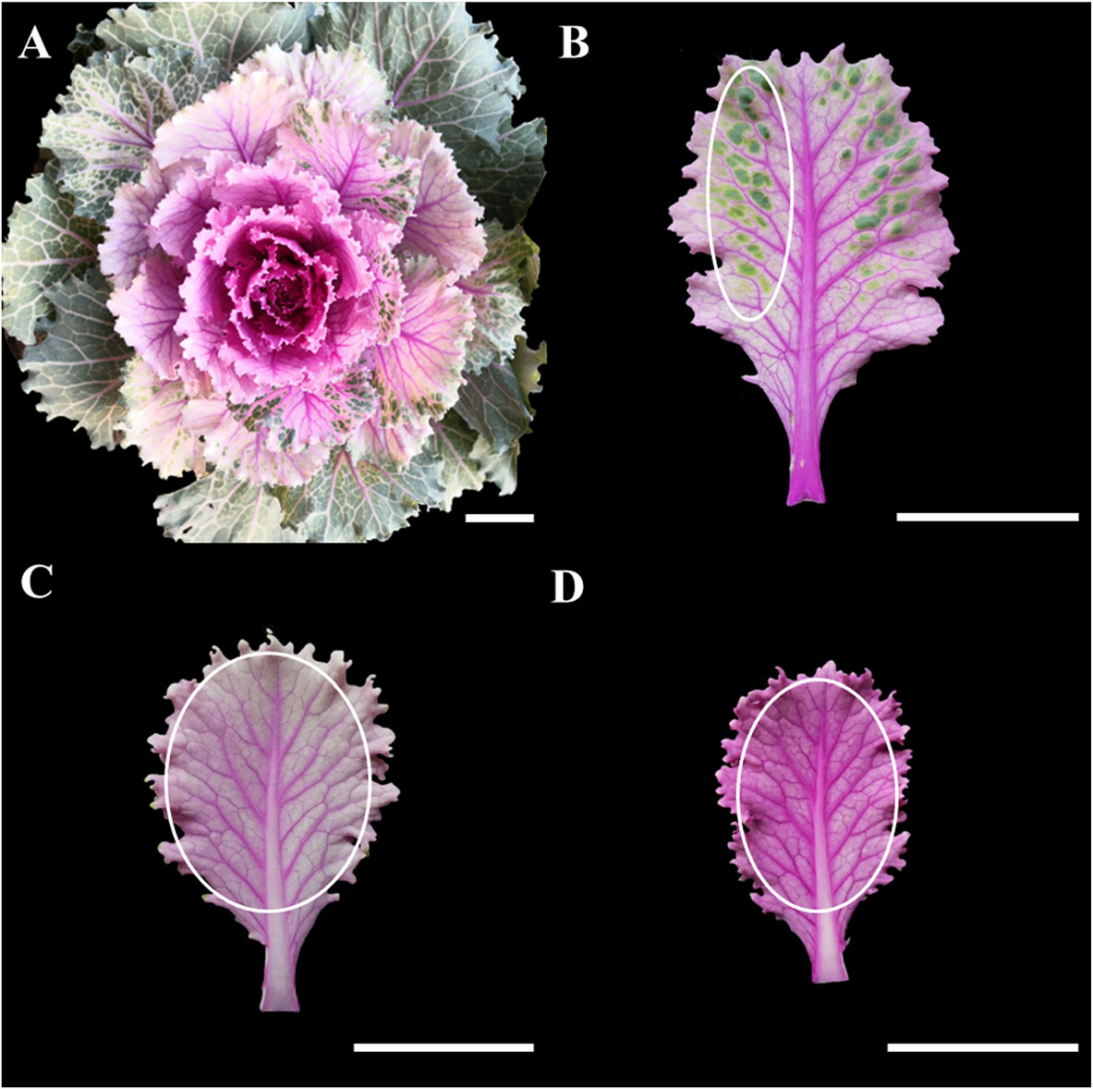
Phenotype and sampled tissues of ‘Pink 42’. **A** The phenotype of ‘Pink 42’. At young stage, leaves are pink, turning into light pink and then variegated pink–green color appears as they developed further. **B** A variegated pink–green leaf of ‘Pink 42’, designated as C1. **C** A light pink leaf of ‘Pink 42’, designated as C2. **D** A pink leaf of ‘Pink 42’, designated as C3. White circles indicate the sampling sites

We have identified an unusual ornamental kale with three different colored leaves (Fig. 1A). The outer leaves are variegated pink–green (Fig. 1B), the middle are light pink (Fig. 1C) and the inner are pink (Fig. 1D) in color. To characterize the differences in gene expression among kale leaves of three different colors (C1, C2, and C3), we constructed cDNA libraries from each leaf type for sequencing. We obtained 413 million raw reads, which were filtered with fastp (https://github.com/OpenGene/fastp) (Table 1) [31]. After removing the reads containing joints, with unknown bases *N* > 10% and low quality, a total of 404 million clean reads were obtained. More than 93% of reads had an average quality score > 30 (Q30 score), and the clean reads ratio was around 98%, indicating that the sequencing data were accurate and reliable for further analysis.

**Table 1:** Reads quality control and comparison of clean reads with the reference genome

Sample	Total Raw Reads (M)	Total Clean Reads (M)	Total Clean Base (Gb)	Q30 (%)	Clean Reads Ratio (%)	Total Mapping (%)	Uniquely Mapping (%)
C1_1	47.74	46.57	6.88	94.88	97.53	90.48	86.52
C1_2	45.18	44.13	6.54	95.12	97.68	91.12	86.82
C1_3	47.63	46.64	6.92	94.5	97.92	90.84	87.27
C2_1	48.93	47.82	7.08	94.79	97.73	88.89	84.48
C2_2	42.53	41.51	6.14	94.69	97.6	89.66	83.85
C2_3	43.71	42.77	6.36	93.93	97.85	88.51	85.67
C3_1	46.49	45.6	6.78	94.9	98.09	90.42	86.9
C3_2	43.65	42.85	6.36	94.9	98.17	90.22	86.73
C3_3	47.37	46.32	6.81	94.91	97.78	90.08	86.79

### Identification and analysis of DEGs

**Fig. 2:**
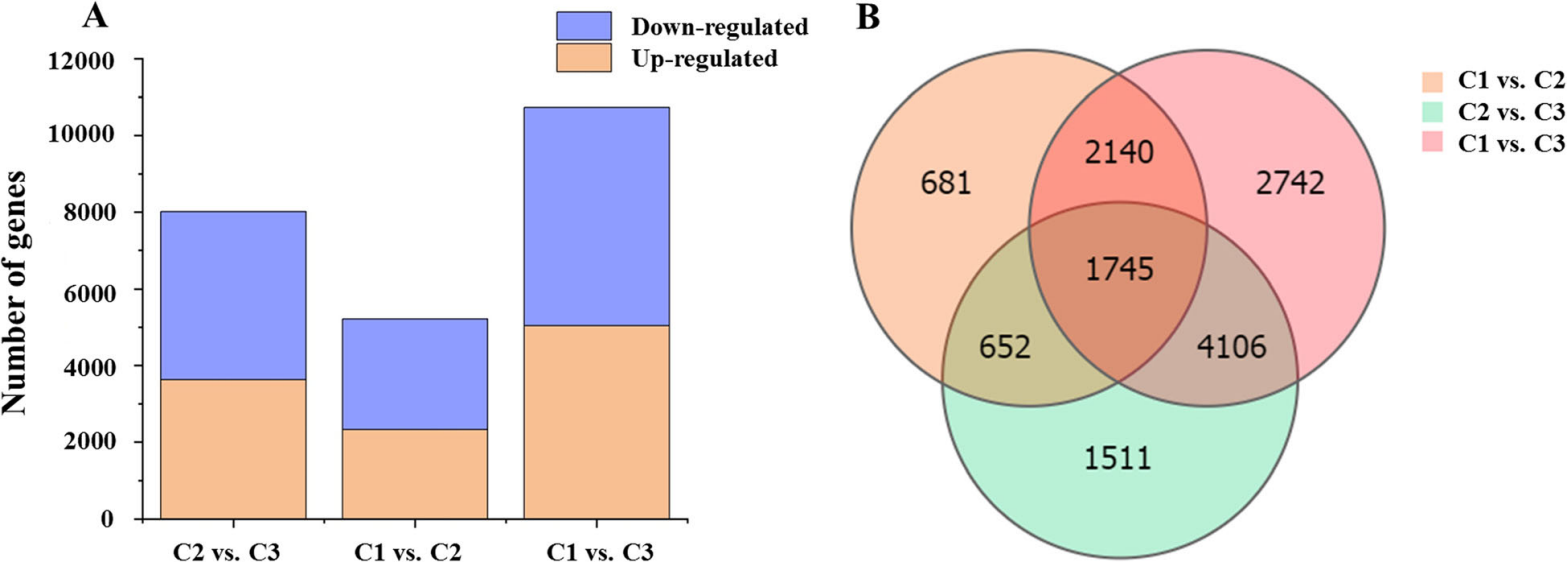
The number of differentially expressed genes (DEGs) detected in comparisons of different colored leaves of ‘Pink 42’. **A** The number of upregulated and downregulated DEGs in pairwise comparisons of variegated pink–green (C1), light pink (C2), and pink (C3) leaves. **B** Venn diagram analysis of DEGs in the pairwise comparisons

DEGs were filtered under a fold–change of more than 2, and a corrected *P*–value ≤0.05, according to DESeq2 [32]. A total of 23,965 DEGs were identified in three comparison groups (C1 versus C2, C1 versus C3, and C2 versus C3). Eight thousand fourteen DEGs were detected in the C2 versus C3 comparison, including 3628 upregulated genes and 4386 downregulated genes (Fig. 2A). Five thousand two hundred eighteen DEGs were detected in the C1 versus C2 comparison, including 2327 upregulated genes and 2891 downregulated genes. There were 10,733 DEGs in the C1 versus C3 comparison, including 5040 upregulated genes and 5693 downregulated genes. As shown in the Venn diagram, there were seven groups present (Fig. 2B). There were 1745 DEGs common to the C1 versus C2, C1 versus C3, and C2 versus C3 comparisons. Two thousand one hundred forty DEGs were common to the C1 versus C2 and C1 versus C3 comparisons. Six hundred fifty-two DEGs were common to the C1 versus C2 and C2 versus C3 comparisons. Four thousand one hundred sixty DEGs were common to the C1 versus C3 and C2 versus C3 comparisons.

### Gene ontology (GO) annotation and DEG enrichment analysis

**Fig. 3:**
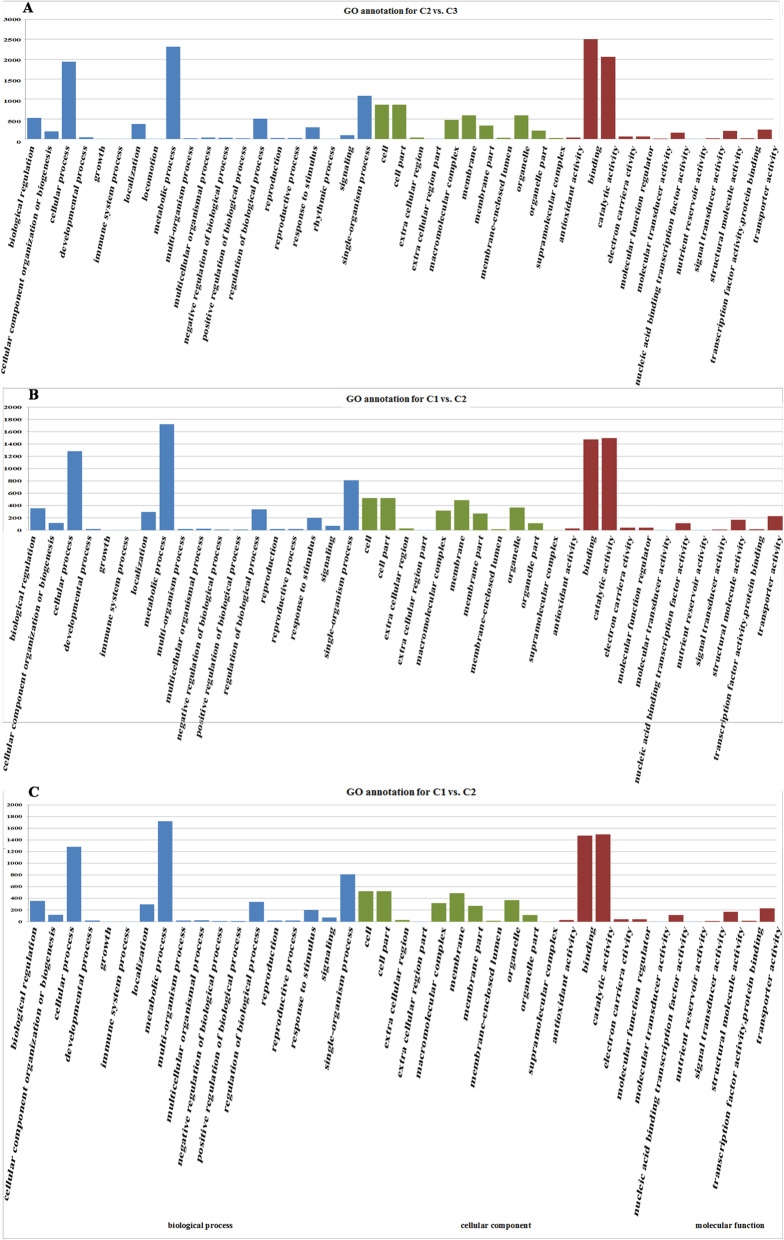
Gene Ontology (GO) annotation of differentially expressed genes (DEGs) detected in comparisons of different colored leaves of ‘Pink 42’. **A** GO annotation of DEGs detected in the C2 versus C3 comparisons. **B** GO annotation of DEGs detected in the C1 versus C2 comparisons. **C** GO annotation of DEGs detected in the C1 versus C3 comparisons

Using Gene Ontology (GO), differentially expressed genes in the three comparisons were classified into three categories: molecular function, cellular component, and biological process. In the C2 versus C3 comparison, 8014 DEGs were classified into 43 GO terms. Of these, 20 belonged to biological process, 11 to cellular component, and 12 to molecular function. ‘Binding’ was the most often enriched term (Fig. 3A). In the C1 versus C2 comparison, 5218 DEGs were classified into 41 GO terms. Of these, 18 belonged to biological process, 11 to cellular component, and 12 to molecular function. ‘Metabolic process’ was the most often enriched term (Fig. 3B). In the C1 versus C3 comparison, 10,733 DEGs were classified into 43 GO terms. Of these, 20 belonged to biological process, 11 terms to cellular component, and 12 to molecular function. “Metabolic process” was the most enriched term (Fig. 3C). The top five enriched terms were ‘metabolic process’, ‘catalytic activity’, ‘binding’, ‘cellular process’, and ‘single–organism process’.

### Kyoto encyclopedia of genes and genomes (KEGG) pathway enrichment analysis of the DEGs

**Fig. 4:**
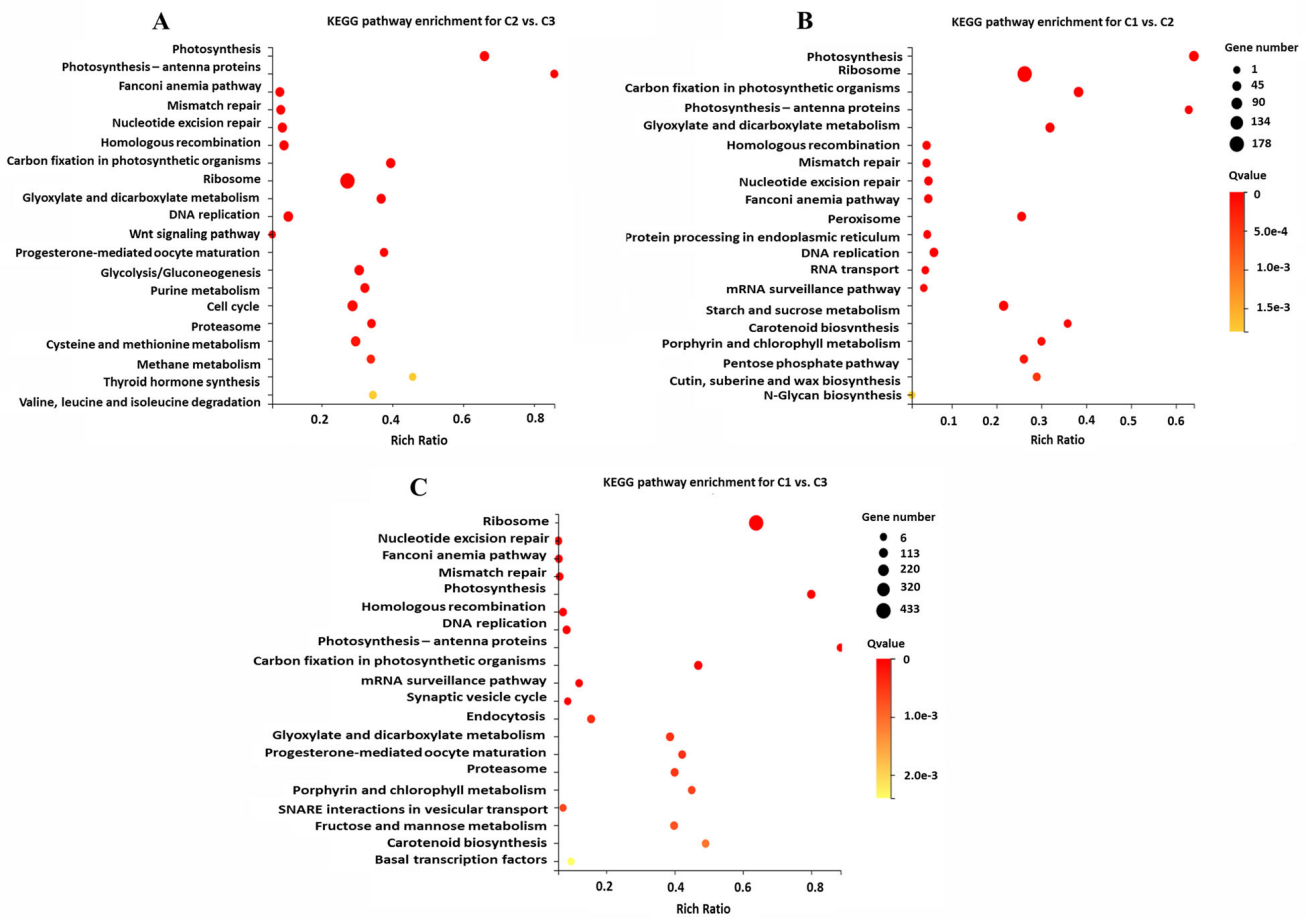
The 20 most enriched Kyoto Encyclopedia of Genes and Genomes (KEGG) pathways from comparisons of different colored leaves. **A** The 20 most enriched KEGG pathways in the C2 versus C3 comparisons. **B** The 20 most enriched KEGG pathways in the C1 versus C2 comparisons. **C** The 20 most enriched KEGG pathways in the C1 versus C3 comparisons

We performed Kyoto Encyclopedia of Genes and Genomes (KEGG) pathway enrichment analysis of the DEGs using the R package. In the C2 versus C3 comparison, ‘photosynthesis’, ‘mismatch repair’, ‘photosynthesis–antenna proteins’, ‘fanconi anemia pathway’, ‘nucleotide excision repair’, and ‘homologous recombination’ were the most significantly enriched pathways (Fig. 4A). In the C1 versus C2 comparison, ‘ribosome’, ‘photosynthesis’, ‘carbon fixation in photosynthetic organisms’, ‘glyoxylate and dicarboxylate metabolism’, ‘porphyrin and chlorophyll metabolism’, and ‘carotenoid biosynthesis’ were the most significantly enriched pathways (Fig. 4B). The six most significantly enriched pathways in the C1 versus C3 comparison were ‘ribosome’, ‘nucleotide excision repair’, ‘fanconi anemia pathway’, ‘mismatch repair’, ‘photosynthesis’, and ‘homologous recombination’ (Fig. 4C).

### Expression patterns of DEGS involved in anthocyanin biosynthesis

**Fig. 5:**
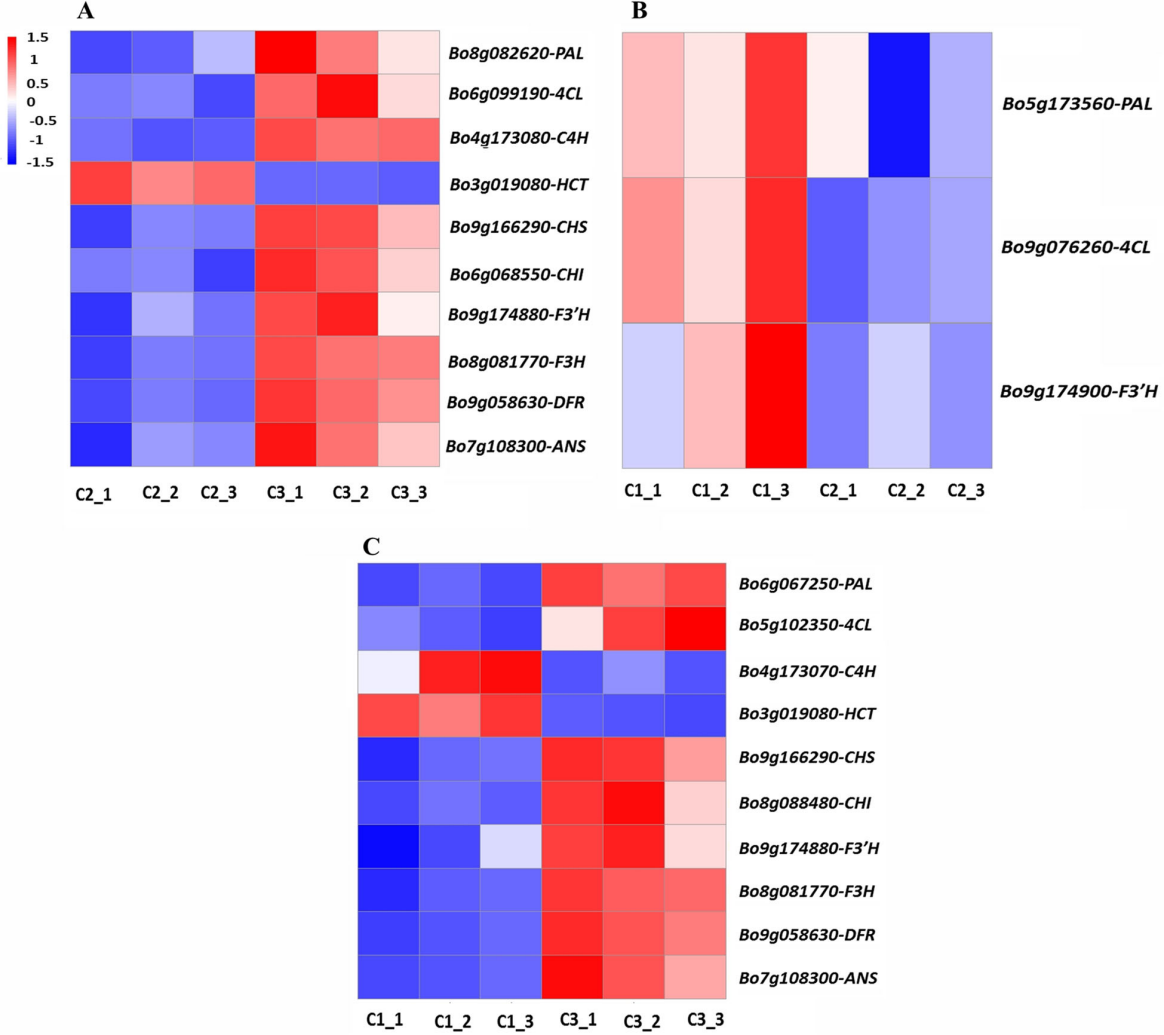
Heatmaps of expression patterns of differentially expressed genes (DEGs) involved in anthocyanin biosynthesis. **A** C2 versus C3. **B** C1 versus C2. **C** C1 versus C3. Red and blue indicate higher and lower FPKM (Fragments Per Kilobase of transcript per Million mapped reads) values, respectively

Across the three comparisons of leaf colors, 15 DEGs related to anthocyanin biosynthesis were detected. Ten anthocyanin biosynthesis–related DEGs were detected in the C2 versus C3 comparison (Fig. 5A). Nine genes were upregulated in C3 compared with C2: *PAL* (*Bo8g082620*), *4CL* (*Bo6g099190*), *C4H* (*Bo4g173080*), *CHS* (*Bo9g166290*), *CHI* (*Bo6g068550*), *F3’H* (*Bo9g174880*), *F3H* (*Bo8g081770*), *DFR* (*Bo9g058630*), and *ANS* (*Bo7g108300*), and one gene, *HCT* (*Bo3g019080*), was downregulated. Of these, *C4H* (*Bo4g173080*) showed the most extreme difference in expression between C2 and C3. *PAL* (*Bo5g137560)*, *4CL* (*Bo9g076260)*, and *F3’H* (*Bo9g174900*), which are involved in the early steps of anthocyanin biosynthesis, were upregulated in variegated pink–green leaves (C1) compared with light pink leaves (C2) (Fig. 5B). *F3’H* (*Bo9g174900*) showed the biggest difference in expression in this comparison. There were 10 DEGs related to anthocyanin biosynthesis in the C1 versus C3 comparison: *PAL* (*Bo6g067250*), *4CL* (*Bo5g102350*), *C4H* (*Bo4g173070*), *HCT* (*Bo3g019080*), *CHS* (*Bo9g166290*), *CHI* (*Bo8g088480*), *F3’H* (*Bo9g174880*), *F3H* (*Bo8g081770*), *DFR* (*Bo9g058630*), and *ANS* (*Bo7g108300*) (Fig. 5C). All of these genes, except for *C4H* and *HCT*, were upregulated in C3 compared with C1. Of these DEGs, *DFR* (*Bo9g058630*) showed the highest expression in pink leaves (C3) compared with variegated pink–green leaves.

### Expression patterns of DEGs related to chlorophyll metabolism

**Fig. 6:**
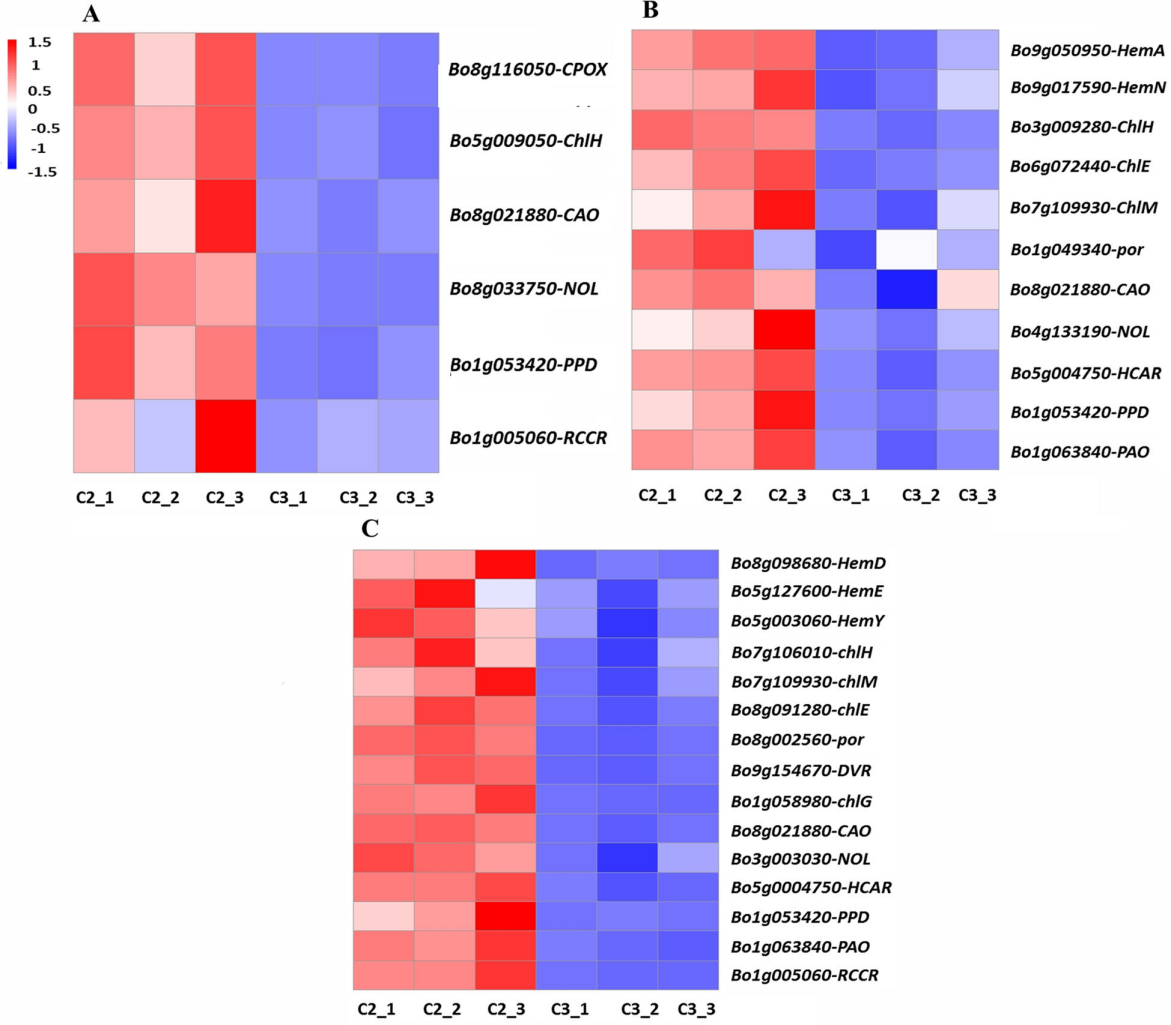
Heatmaps of expression patterns of differentially expressed genes (DEGs) involved in anthocyanin biosynthesis. **A** C2 versus C3. **B** C1 versus C2. **C** C1 versus C3. Red and blue indicate higher and lower FPKM (Fragments Per Kilobase of transcript per Million mapped reads) values, respectively

Two DEGs related to chlorophyll biosynthesis (*CPOX*, *Bo8g116050*; *ChlH*, *Bo5g009050*), two related to the chlorophyll cycle (*CAO*, *Bo8g021880*; *NOL*, *Bo8g033750*), and two related to chlorophyll degradation (*PPD*, *Bo1g053420*; *RCCR*, *Bo1g005060*) were detected in the C2 versus C3 comparison (Fig. 6A). All of these DEGs were upregulated in light pink leaves (C2) compared with pink leaves (C3). Of these DEGs, *PPD* (*Bo1g053420*) showed the highest relative expression level in light pink leaves. In the C1 versus C2 comparison, six DEGs involved in chlorophyll biosynthesis (*HemA*, *Bo9g050950*; *HemN*, *Bo9g017590*; *chlH*, *Bo3g009280*; *chlM*, *Bo7g109930*; *chlE*, *Bo6g072440*; *por*, *Bo1g049340*), three involved in the chlorophyll cycle (*CAO*, *Bo8g021880*; *NOL*, *Bo4g133190*; *HCAR*, *Bo5g004750*), and two involved in chlorophyll degradation (*PPD*, *Bo1g053420*; *PAO*, *Bo1g063840*) were detected (Fig. 6B). All of these DEGs were upregulated in variegated pink–green leaves (C1) relative to light pink leaves (C2). Compared with C2, *PPD* (*Bo1g053420*) showed the highest expression in variegated pink–green leaves (C1). In the C1 versus C3 comparison, we detected nine DEGs related to chlorophyll biosynthesis (*HemD*, *Bo8g098680*; *HemE*, *Bo5g127600*; *HemY*, *Bo5g003060*; *chlH*, *Bo7g106010*; *chlM*, *Bo7g109930*; *chlE*, *Bo8g091280*; *por*, *Bo8g002560*; *DVR*, *Bo9g154670*; *chlG*, *Bo1g058980*), three involved in the chlorophyll cycle (*CAO*, *Bo8g021880*; *NOL*, *Bo3g003030*; *HCAR*, *Bo5g004750*) and three involved in chlorophyll degradation (*PPD*, *Bo1g053420*; *PAO*, *Bo1g063840*; *RCCR*, *Bo1g005060*) (Fig. 6C). All of these DEGs were downregulated in pink leaves (C3) relative to variegated pink–green leaves (C1). Of these DEGs, *PPD* (*Bo1g053420*) showed the highest expression in C1 relative to C3.

### Expression patterns of DEGs related to carotenoid biosynthesis

**Fig. 7:**
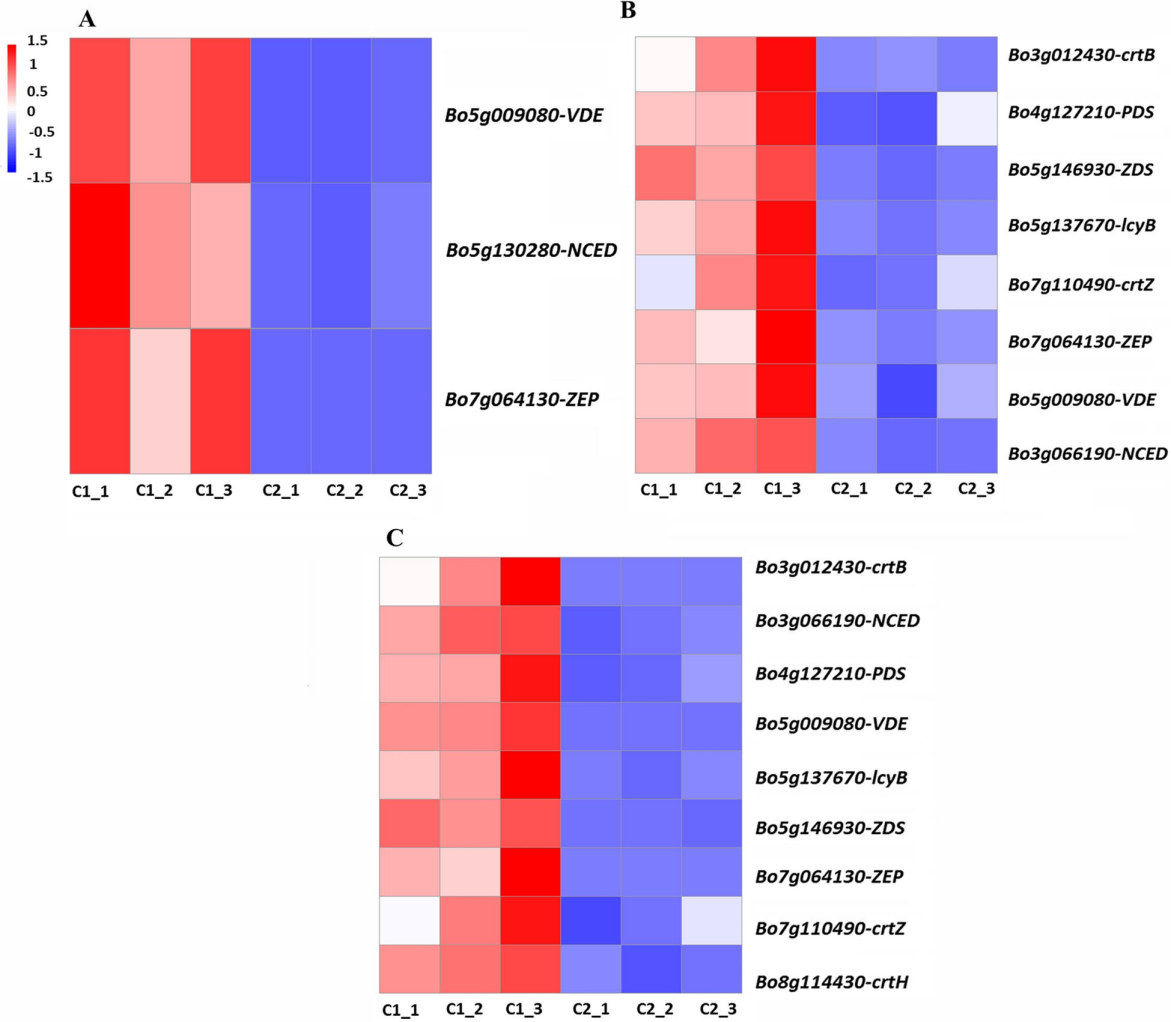
Heatmaps of expression patterns of differentially expressed genes (DEGs) involved in anthocyanin biosynthesis. **A** C2 versus C3. **B** C1 versus C2. **C** C1 versus C3. Red and blue indicate higher and lower FPKM (Fragments Per Kilobase of transcript per Million mapped reads) values, respectively

Three DEGs involved in carotenoid biosynthesis (*ZEP*, *Bo7g064130*; *VDE*, *Bo5g009080*; *NCED*, *Bo5g130280*) were detected in the C2 versus C3 comparison (Fig. 7A). All of these DEGs were upregulated in light pink leaves (C2) relative to pink leaves (C3). *VDE* (*Bo5g009080*) showed the highest expression in C2 compared to C3. Eight DEGs involved in carotenoid biosynthesis (*crtB*, *Bo3g012430*; *PDS*, *Bo4g127210*; *ZDS*, *Bo5g146930*; *lcyB*, *Bo5g137670*; *crtZ*, *Bo7g110490*; *ZEP*, *Bo7g064130*; *VDE*, *Bo5g009080*; *NCED*, *Bo3g066190*) were detected in the C1 versus C2 comparison (Fig. 7B). All of these DEGs were upregulated in variegated pink–green leaves (C1) relative to light pink leaves (C2). *crtB* (*Bo3g012430*) showed the biggest difference in expression in this comparison. In the C1 versus C3 comparison, we detected nine DEGs involved in carotenoid biosynthesis (*crtB*, *Bo3g012430*; *PDS*, *Bo4g127210*; *ZDS*, *Bo5g146930*; *crtH*, *Bo8g114430*; *lcyB*, *Bo5g137670*; *crtZ*, *Bo7g110490*; *ZEP*, *Bo7g064130*; *VDE*, *Bo5g009080*; *NCED*, *Bo3g066190*) (Fig. 7C). All of these DEGs were upregulated in variegated pink–green leaves (C1) compared to pink leaves (C3).

### Identification and expression patterns of DEGs encoding transcription factors (TFs)

Transcription factors (TFs) were identified by aligning the DEGs to a plant TF database with BLASTX (http://planttfdb.cbi.pku.edu.cn/). Four hundred eighty-three TFs including 102 MYB, 62 bHLH, and 45 WD40 TFs were identified in the C2 versus C3 comparison (Additional file 1: Table S1). *MYB12* (*Bo4g004500*), *MYB113* (*Bo9g100940*), *MYB113* (*Bo9g099880*), *MYB3* (*Bo7g057770*), *MYB48* (*Bo3g130340*), and *MYBL2* (*Bo2g070770*) were annotated as involved in the regulation of anthocyanin biosynthesis. *MYB12* and *MYBL2* showed the most extreme difference in expression between C2 and C3. *MYB12* (*Bo4g004500*) was upregulated in C3, while *MYBL2* (*Bo2g070770*) upregulated in C2. Among the *bHLH* genes, four were annotated as involved in the regulation of anthocyanin biosynthesis: *EGL3* (*Bo9g035460*), *EGL3* (*Bo9g029230*), *GL3* (*Bo4g141990*), and *TT8* (*Bo9g086910*). In addition, we identified seven TFs involved in the regulation of chlorophyll metabolism, including two *bZIP* genes (*ABF4*, *Bo3g073080*; *ABF4*, *Bo1g114840*), two *C2C2–GATA* genes (*GATA21*, *Bo9g127360*; *GATA21*, *Bo3g019610*), two *ARR–B* genes (*GLK3*, *Bo7g003410*; *GLK2*, *Bo7g067200*), and one *bHLH* gene (*PIL5*, *Bo7g003890*). *GLK2* (*Bo7g067200*) showed the most extreme difference in expression between C2 and C3.

There are 159 TFs including 37 MYBs, 49 bHLHs, and 18 WD40s in the C1 versus C2 comparison (Additional file 1: Table S2). Four *bHLH* genes (*TT8*, *Bo9g086910*; *MYC4*, *Bo1g017990*; *MYC3*, *Bo7g075710*; *MYC2*, *Bo5g086990*) were annotated as involved in the regulation of anthocyanin biosynthesis. The gene *TT8* showed the most extreme difference in expression between C1 and C2. Two genes encoding C2C2–GATA TFs were detected in the C1 versus C2 comparison (*GATA21*, *Bo2g033970*; *GATA22*, *Bo1g042950*). The gene *GATA22* showed the most extreme difference in expression between C1 and C2. Interestingly, the gene *RR1* (*Bo3g068530*) identified in this comparison was not only involved in the regulation of anthocyanin biosynthesis but also in the regulation of chlorophyll metabolism. Its expression was highest in C2 compared with both C3 and C1.

### Quantitative real–time PCR (qRT–PCR) analysis of candidate genes

**Fig. 8:**
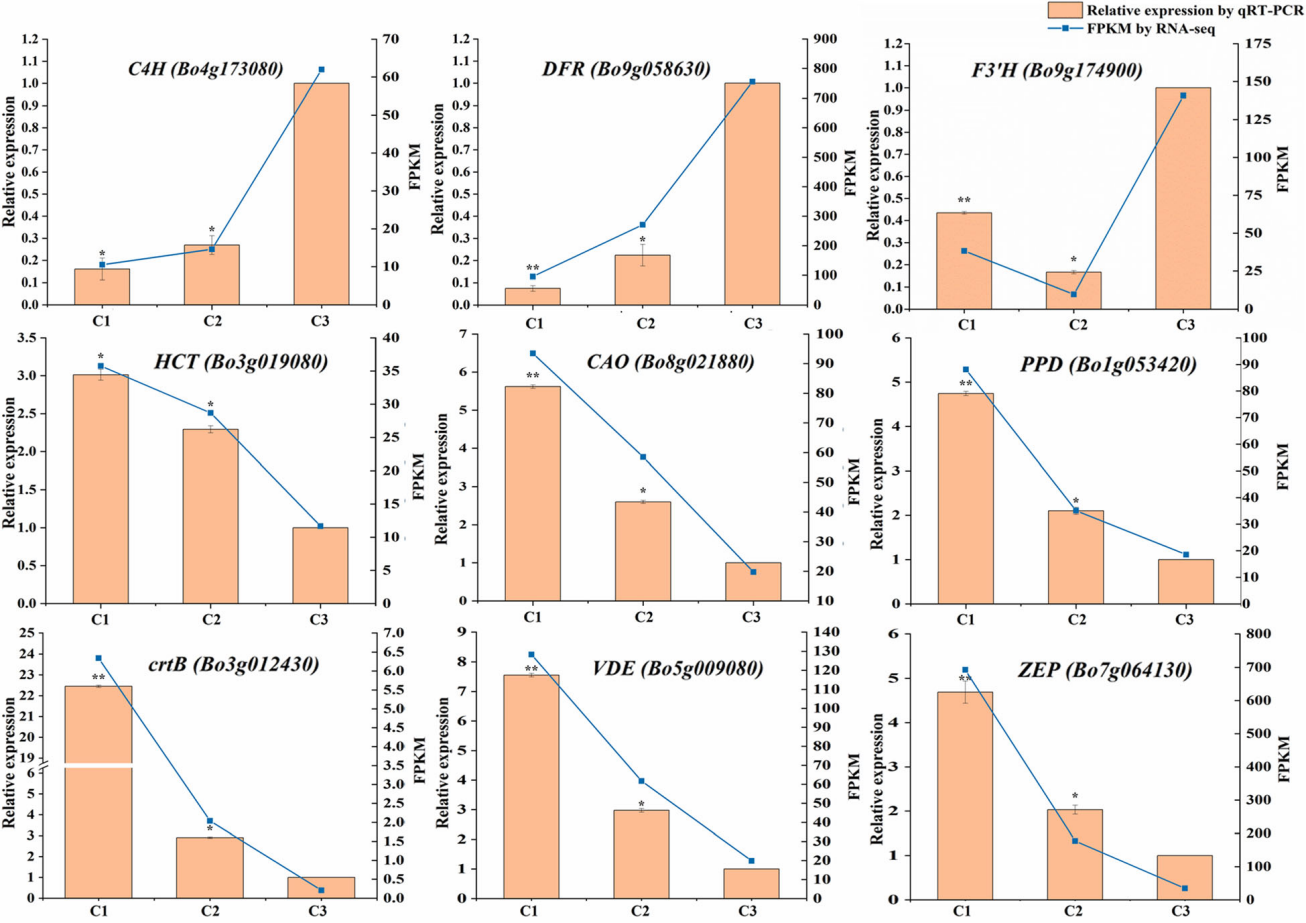
Relative expression level and FPKM (Fragments Per Kilobase of transcript per Million mapped reads) of candidate structural genes involved in anthocyanin biosynthesis, chlorophyll metabolism, and carotenoid biosynthesis in three different colored leaves. C1, C2, and C3 stands for variegated pink–green leaves, light pink leaves, and pink leaves respectively. C3 was used as reference to calculate the relative expression of genes. Error bar represents ±SE of the means of three duplications. Asterisks represent significant differences by T–test (*p < 0.05, ** *p* < 0.01)

**Fig. 9:**
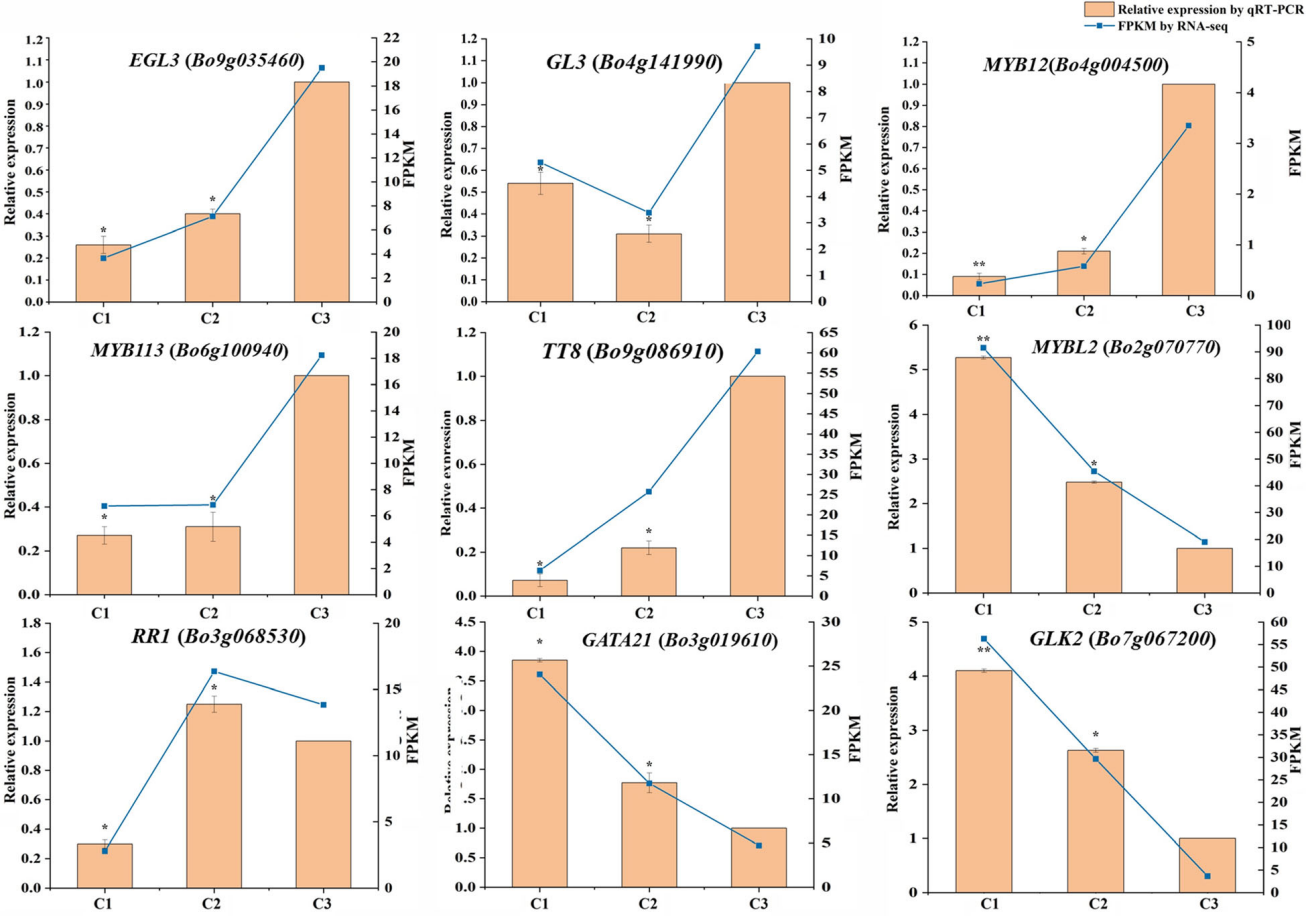
Relative expression level and FPKM (Fragments Per Kilobase of transcript per Million mapped reads) of candidate regulatory genes involved in anthocyanin biosynthesis, chlorophyll metabolism, and carotenoid biosynthesis in three different colored leaves. C1, C2, and C3 stands for variegated pink–green leaves, light pink leaves, and pink leaves respectively. C3 was used as reference to calculate the relative expression of genes. Error bar represents ±SE of the means of three duplications. Asterisks represent significant differences by T–test (*p < 0.05, ** *p* < 0.01)

Nine DEGs encoding structural genes and nine DEGs encoding regulatory genes were selected for qRT–PCR test. The results confirmed that the selected genes were differentially expressed in three different colored leaves. Moreover, the most genes involved in anthocyanin biosynthesis were downregulated, while the genes involved in chlorophyll metabolism and carotenoid biosynthesis were upregulated in the transition from pink leaf to variegated pink–green leaf. It was coincided with the results obtained through RNA-seq. The gene *DFR* (*Bo9g058630*), involved in anthocyanin biosynthesis, was not only downregulated during the pink leaf (C3) changing into variegated pink–green (C1) but also showed a biggest difference between C3 and C1. The gene *HCT* (*Bo3g019080*) which involved in anthocyanin biosynthesis in a reverse way was upregulated from C3 to C1. In addition, the genes *CAO* (*Bo8g021880*) and *PPD* (*Bo1g053420*), which involved in chlorophyll metabolism, and genes *crtB* (*Bo3g012430*), *VDE* (*Bo5g009080*) and *ZEP* (*Bo7g064130*), which involved in carotenoid biosynthesis, were all upregulated from C3 to C1 (Fig. 8). The genes *EGL3* (*Bo9g035460*), *TT8* (*Bo9g086910*), *MYB113* (*Bo6g100940*) and *MYB12* (*Bo4g004500*), positively regulating anthocyanin biosynthesis, were downregulated from C3 stage to C1 stage (Fig. 9). *MYBL2* (*Bo2g070770*), a gene negatively regulating anthocyanin biosynthesis, was upregulated from C3 stage to C1 stage. The expression of gene *RR1* (*Bo3g068530*) was highest at C2 stage. The fold change of genes expression regulating anthocyanin biosynthesis was larger in C2 versus C3 than C1 versus C2 comparison. Instead, the fold change of genes expression regulating chlorophyll biosynthesis was larger in C1 versus C2 than C2 versus C3 comparison (Fig. 9).

### Anthocyanin, chlorophyll, and carotenoid contents in three different colored leaves

**Fig. 10:**
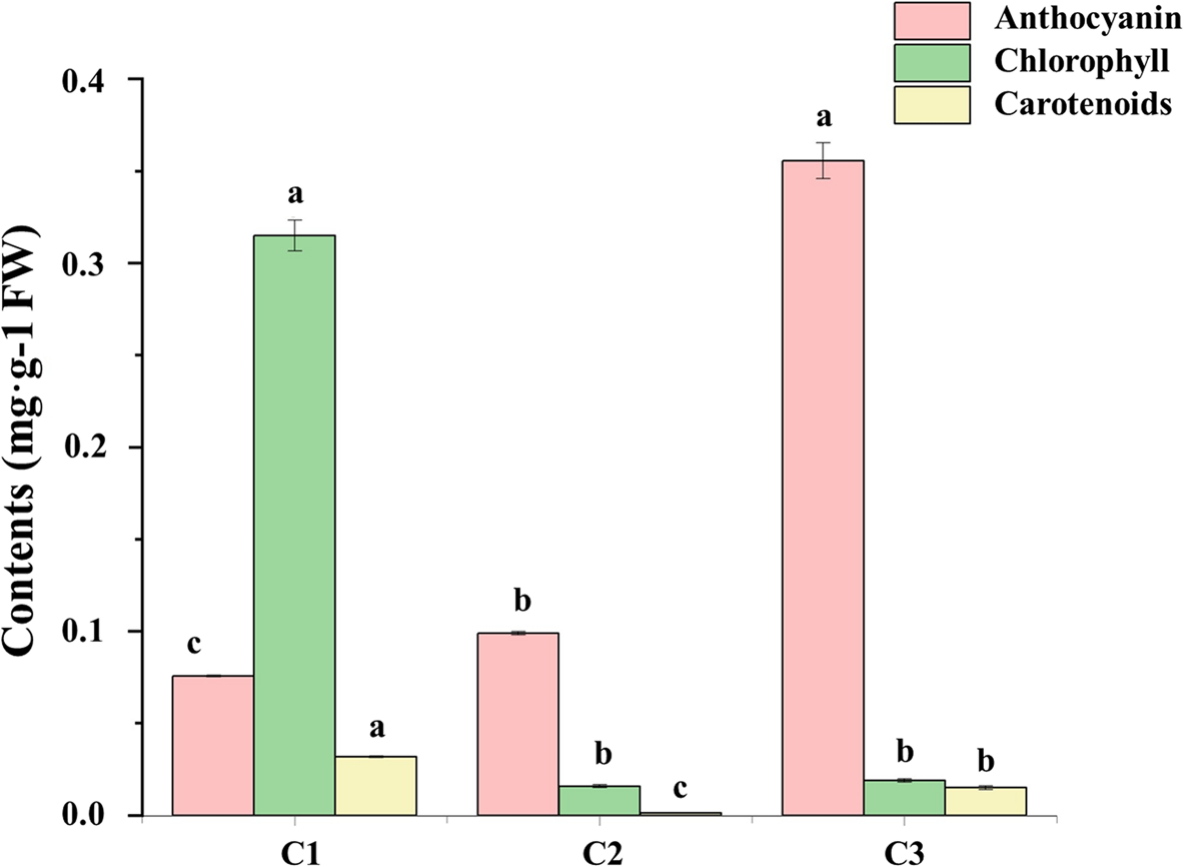
Anthocyanin, chlorophyll, and carotenoid contents in different colored leaves of ‘Pink 42’. C1, C2, and C3 stands for variegated pink–green leaves, light pink leaves, and pink leaves, respectively. Different letters indicate statistically significant differences in the contents of the same pigment among different leaf types (*p* < 0.05) based on the analysis of variance (ANOVA) (Tukey test)

We quantified anthocyanin, chlorophyll, and carotenoid contents in leaves of three different colors. The contents of anthocyanin, chlorophyll, and carotenoid in C3 were 0.3555 mg·g^− 1^ (fresh weight; FW), 0.0189 mg·g^− 1^ (FW), and 0.0150 mg·g^− 1^ (FW), respectively. C2 leaves had 0.0988 mg·g^− 1^ (FW) anthocyanin, 0.0158 mg·g^− 1^ (FW) chlorophyll, and 0.0012 mg·g^− 1^ (FW) carotenoid. The contents of anthocyanin, chlorophyll, and carotenoid in C1 were 0.0755 mg·g^− 1^ (FW), 0.3148 mg·g^− 1^ (FW), and 0.0318 mg·g^− 1^ (FW), respectively. These results showed that pink leaves had the highest anthocyanin content. Chlorophyll and carotenoid contents were lowest in the light pink leaves. Chlorophyll and carotenoid contents were highest and anthocyanin content was lowest in the variegated pink–green leaves (Fig. 10).

## Discussion

Leaf color is one of the most important traits of ornamental kale. Pink–leaved ornamental kale with green variegation is a unique material for exploring the molecular mechanism of leaf color formation. In this study, we used RNA sequencing to analyze the expression patterns of DEGs involved in anthocyanin and carotenoid biosynthesis, and chlorophyll metabolism, in different colored ornamental kale leaves. The contents of anthocyanin, chlorophyll, and carotenoids were analyzed in parallel. The results revealed the relationship between differences in gene expression and the variation of pigment contents, allowing us to identify candidate genes involved in pigment biosynthesis and metabolism.

### Differential expression of genes related to anthocyanin biosynthesis

In recent years, transcriptome sequencing technology has been widely used to identify candidate genes and analyze gene expression because of its low cost and high throughput [33]. A previous comparative transcriptome analysis identified 81 anthocyanin biosynthetic genes in the *B. oleracea* reference genome. Of these genes, *BoDFR1*, *BoANS*, and *BoUGT79B1.1*, *BoTTG1*, *BoTT8*, *BoMYBL2.1*, *BoTT19.1*, and *BoTT19.2* are important for determining red versus white pigmentation in ornamental kale [28]. *LBD39*, *LBD37*, and *C4H* were associated with anthocyanin biosynthesis in bicolor ornamental kale [29]. *BoC4H2*, *BoUGT9*, and *BoGST21* have been proposed as key genes for anthocyanin biosynthesis in purple ornamental kale compared with white [30]. A comparative analysis of purple and green ornamental kale identified 46 DEGs involved in anthocyanin biosynthesis, of which 45 were upregulated in purple leaves [30].

In the current study, 15 DEGs related to anthocyanin biosynthesis were detected in three pairwise comparisons of different colored leaves of the ‘Pink 42’ cultivar of ornamental kale. It was noted that all of the DEGs related to earlier anthocyanin biosynthesis in the C1 (variegated pink–green leaves) versus C2 (light pink leaves) comparison (*PAL*, *Bo5g137560*; *4CL*, *Bo9g076260*; *F3’H*, *Bo9g174900*) were upregulated in C2, but anthocyanin content was lower in C2 than in C1. Because the expression pattern of these genes is contrary to the trend in pigment variation, we can infer that they are not key genes for controlling anthocyanin biosynthesis. Liu et al. identified *Bo9g058630*, which is homologous to *AT5G42800* (*DFR*), the structural gene for dihydroflavonol 4–reductase, as a key gene for anthocyanin biosynthesis in ornamental kale [26]. Similar results were reported by Ren et al. [27]. Cinnamic acid–4–hydroxylase (*C4H*), a key enzyme in phenylpropanoid biosynthesis, affects the early steps of anthocyanin biosynthesis. In our comparison of variegated pink–green leaves (C1) with pink leaves (C3), *DFR* (*Bo9g058680*) was the most downregulated DEG in C1. In C2 (light pink) versus C3 (pink), *C4H* (*Bo4g173080*) was the most downregulated DEG. We got similar results in qRT–PCR test. These results indicate that decreased expression of *DFR* (*Bo9g058680*) and *C4H* (*Bo4g173080*) results in decreased anthocyanin content as leaves develop.

Our study also revealed a branched pathway for anthocyanin biosynthesis. Unlike the pathway reported previously, this one synthesizes anthocyanin through the formation of caffeoyl–CoA from p–coumaroyl CoA by the action of shikimate O–hydroxycinnamoyltransferase (*HCT*), followed by steps catalyzed by enzymes encoded by *CHS*, *F3H, DFR*, and *ANS*. In *A. thaliana*, *HCT* is a lignin biosynthetic gene. Silencing of *HCT* in plants inhibits lignin biosynthesis, leading to activation of *CHS* and the accumulation of several flavonol glycosides and acylated anthocyanin [34]. In the current study, *HCT* (*Bo3g019080*) was detected as a DEG in comparisons between C1 versus C3 and C2 versus C3. Furthermore, the expression of *HCT* was downregulated in C3 compared with both C1 and C2, indicating that anthocyanin accumulation is associated with downregulation of *HCT* (*Bo3g019080*) in ornamental kale.

### DEGs related to chlorophyll metabolism and carotenoid biosynthesis

Previously, it was shown that decreasing chlorophyll content in triple–color ornamental kale leaves is caused by chlorophyll biosynthesis inhibition [24]. In our comparisons of C1 versus C2 and C2 versus C3, all DEGs related to chlorophyll metabolism, including chlorophyll biosynthesis, chlorophyll cycle, and chlorophyll degradation, were upregulated in C2 compared with C1 and C3 compared with C2. This result indicates that both chlorophyll biosynthesis and degradation are upregulated in the transition from pink to variegated pink–green leaf pigmentation. This is contrary to our expectation that an increased rate of biosynthesis and a decreased rate of degradation would be responsible for the accumulation of chlorophyll. *CAO* (*Bo8g021880*), a DEG related to chlorophyll cycle, was identified in three comparisons. The expression of gene *CAO* was upregulated during the pink leaves changed to the variegated pink–green revealed through qRT–PCR experiment. *PPD* (*Bo1g053420*), which is related to chlorophyll degradation, was not only identified as a DEG in all three comparisons, but also showed the biggest differences in expression level among the DEGs. We could infer that genes *CAO* and *PPD* may play important roles in chlorophyll metabolism in ornamental kale. *VDE* (*Bo5g009080*) and *ZEP* (*Bo7g064130*), which are related to carotenoid biosynthesis, were also detected as DEGs across all three comparisons. In addition, *crtB* (*Bo3g012430*), another carotenoid biosynthesis gene, showed the highest expression in variegated pink–green leaves relative to the other samples. These results suggest that carotenoid biosynthesis might be closely related to the expression of *VDE* (*Bo5g009080*), *ZEP* (*Bo7g064130*), and *crtB* (*Bo3g012430*) in ornamental kale leaves.

### Leaf color is closely related to pigment content

Anthocyanins, chlorophyll, and carotenoids are important pigments in ornamental kale. The concentration ratios of these pigments strongly influence the color of kale leaves [25]. As pink leaves became light pink, the anthocyanin and carotenoid contents declined, while the chlorophyll content decreased slightly, but not significantly. As light pink leaves subsequently became variegated pink–green, the anthocyanin content was continued to decline, while the chlorophyll and carotenoid contents increased, ultimately producing green variegation. As the leaves developed, chlorophyll and carotenoids were first degraded and then accumulated. The simultaneous increase in chlorophyll and decrease anthocyanin leads to the formation of variegated pink–green leaves.

## Conclusions

**Fig. 11:**
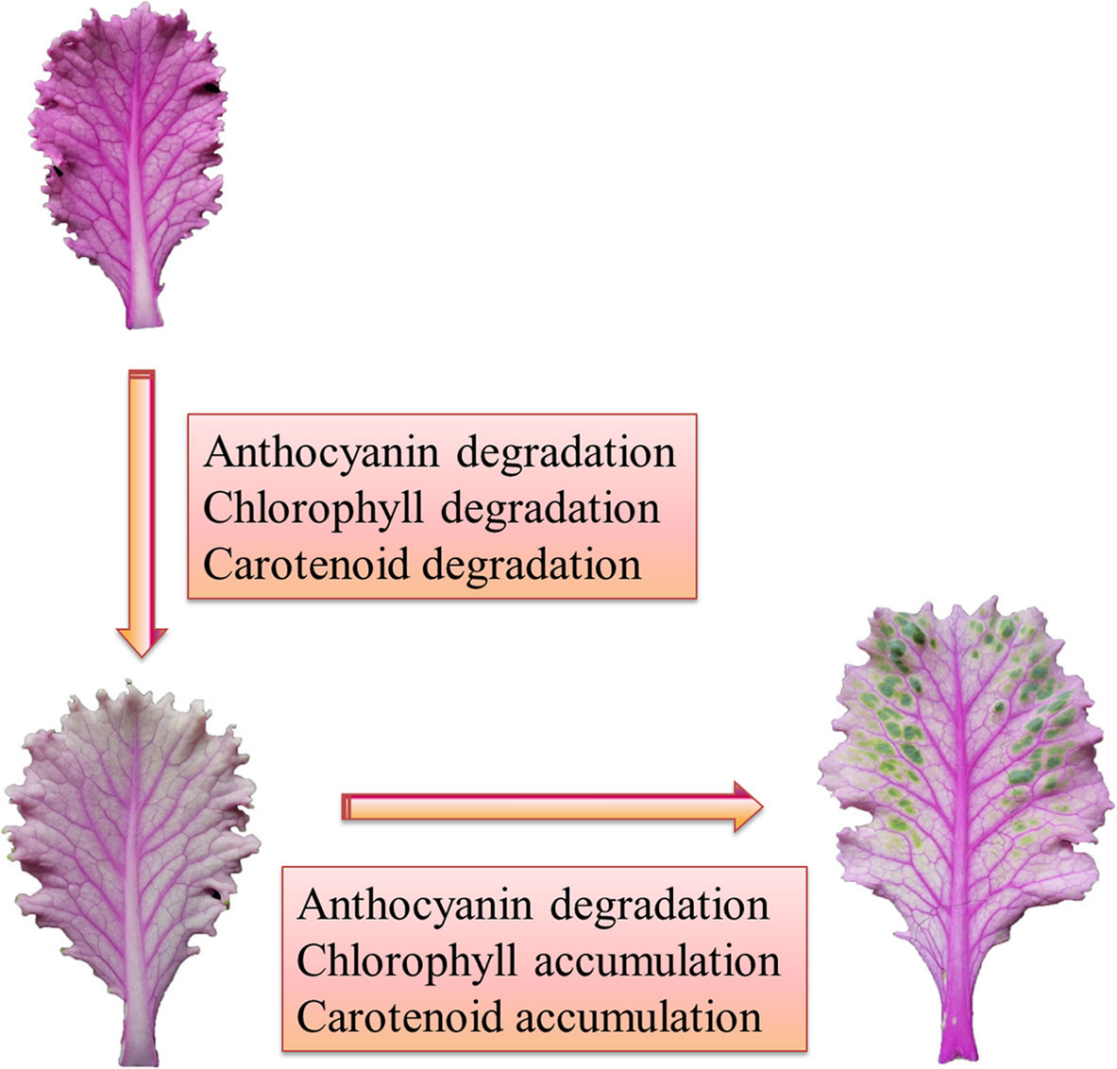
Tentative model of the mechanism underlying pigments change occurring as kale leaves change from pink to variegated pink–green coloration

In this study, transcriptome sequencing and pigment content analysis revealed that DEGs involved in anthocyanin biosynthesis were downregulated in light pink leaves (C2) compared with pink leaves (C3), while anthocyanin content was lower in C3 than C2 leaves. Similar results were obtained in the C2 versus C1 (variegated pink–green) comparison. In addition, DEGs involved in chlorophyll metabolism and carotenoid biosynthesis were upregulated in C1 compared with C3, while chlorophyll and carotenoid contents were increased in C3 compared with C1. We propose that the changes in leaf color from pink to light pink to light pink with green variegation are caused by continuous anthocyanin degradation and chlorophyll accumulation, along with the successive degradation and accumulation of carotenoid (Fig. 11). These findings provide a basis for exploring the molecular mechanism of variegation and color development in ornamental kale leaves.

## Methods

### Plant materials

Fresh leaves from the rosette stage of ornamental kale (*Brassica oleracea var. acephala* DC) cultivar ‘Pink 42’ were used for RNA sequencing, qRT–PCR experiment, and pigment content analysis. All experimental materials were cultivated in a greenhouse at 25 °C ± 3 °C under a 16 h light/8 h dark photoperiod at Shenyang Agricultural University, Shenyang, China, in 2019. The variegated pink–green leaves were named C1, the light pink leaves C2, and the pink leaves C3 (Fig. 1). Three independent biological replicates were conducted for each experiment. The ornamental kale cultivar ‘Pink 42’ used in the current study was identified by Prof. Pengfang Zhu. It is stored in the Shenyang Agricultural University Ornamental Kale Germplasm Garden. The plant materials are available from the corresponding author on reasonable requests.

### RNA extraction

Total RNA was extracted using RNAiso reagent (TaKaRa, Japan), according to the manufacturer’s instructions. The quality and purity of the extracted RNA were measured with an agarose gel and NanoDrop 8000 spectrophotometer (Thermo Scientific, USA). RNA integrity was evaluated using a 2100 Bioanalyzer RNA Nano chip device (Agilent, USA).

### Library construction and sequencing

The Arraystar Seq–Star™ Rapid RNA–seq Kit (Illumina) was used to construct cDNA libraries according to the manufacturer’s instructions. Briefly, total RNA was purified and fragmented, and the random N6 primers were reverse–transcribed for first–strand biosynthesis. Then, the cDNA double strand was synthesized to form double–stranded DNA using DNA polymerase Ι and RNase H. The double–stranded DNA was used as a template for PCR amplification to construct the final DNA library. Finally, the cDNA libraries were sequenced on the Illumina HiSeqTM 2000 platform (Gooalgene Technology Co., Ltd., Wuhan, China).

### RNA sequencing and data analysis

Sequencing data (raw reads) containing joints, reads with unknown bases *N* > 10%, and reads of low quality, were filtered with fastp (https://github.com/OpenGene/fastp) [31]. Filtered clean reads were then aligned to the *B. oleracea* reference genome (http://plants.ensembl.org/Brassica_oleracea/Info/Index) using HISAT [35]. DEGs were detected using DEGseq2. We identified DEGs with a fold–change of two or more, and a Q–value of ≤0.05 [32, 36]. Gene functional annotation, classification and pathway enrichment analysis were performed using Gene Ontolog (http://geneontology.org/docs/ontology–documentation/) and the Kyoto Encyclopedia of Genes and Genomes (KEGG) (https://www.kegg.jp/kegg/pathway.html). Enrichment analysis and the determination of *P*–values were carried out using the phyper function in R, with subsequent false discovery rate (FDR) correction for the *P*–value. A Q–value of < 0.05 was considered a significant enrichment.

### qRT–PCR validation of candidate gene expression

To verify the reliability of the RNA–seq results, nine candidate genes were selected for Quantitative Real–time PCR analysis, including four anthocyanin biosynthesis genes (*C4H*, *Bo4g173080*; *F3’H*, *Bo9g174900*; *DFR*, *Bo9g058630*; and *HCT*, *Bo3g019080*), two chlorophyll metabolism genes (*PPD*, *Bo1g053420*; and *CAO*, *Bo8g021880*), and three carotenoid biosynthesis genes (*VDE*, *Bo5g009080*; *ZEP*, *Bo7g064130*; and *crtB*, *Bo3g012430*). The gene *BoACTIN* (*Bo5g117040*) was used as an internal control. C3 was used as reference to calculate the relative expression of genes. Quantitative Real–time PCR was performed in a total volume of 20 μL reaction system containing 10 μL 2 TB Green Premix Ex Taq II (Takara, Dalian, China), 0.4 μL 50 ROX Reference Dye, 2 μL of 50 ng/μL cDNA, 1 μL of 10 μM gene*–*specific primer (Additional file 2: Table S3), and 5.6 μL RNase–free water. The reaction conditions were set at 95 °C for 30 s, 40 cycles of 95 °C for 5 s, 60 °C for 30 s. The data were analyzed using StepOnePlus Real–Time PCR System (Applied Biosystems, America). Three independent biological and technical replicates were conducted for each experiment. The relative expression levels of the candidate genes were determined using the 2^*−* ΔΔ*CT*^ method [37].

### Determination of anthocyanin, chlorophyll, and carotenoid contents

Anthocyanin, chlorophyll, and carotenoid contents were extracted and determined as previously described [25].

Briefly, anthocyanin content was determined as follows. Fresh leaf samples (2.0 g) were added to 30 mL of extraction solution (0.1 mol/L HCl, 95% ethanol = 1: 1). Samples were extracted in a water bath at 60 °C for 1 h, centrifuged at 5000 rpm/min at 4 °C for 15 min, and then the supernatant was collected. Two 1–mL aliquots of anthocyanin were collected, added to 2 mL of 1 mol/L KCl buffer at pH 1.0 and 2 mL of 1 mol/L sodium acetate buffer at pH 4.0, and shaken well. The absorbance value at 520 nm and 700 nm was determined. Three independent technical replicates were conducted for all the experiments. Anthocyanin content was determined using the following equations:

$$ {\displaystyle \begin{array}{c}\mathrm{A}=\left(\mathrm{A}520-\mathrm{A}700\right)\ \mathrm{pH}\ 1.0-\left(\mathrm{A}520-\mathrm{A}700\right)\ \mathrm{pH}\ 4.5\\ {}\mathrm{C}\left(\mathrm{mg}\cdot \mathrm{g}-1\right)=\frac{A\times V\times n\times M}{\varepsilon \times m}\end{array}} $$

A, the difference of the absorbance value at pH 1.0 and pH 4.5 under 520 nm and 700 nm; V, total volume of extract (mL); n: dilution time; M, the molecular mass of cyanidin–3–glucoside chloride (449.2); ε, the molar absorptivity of cy–3–glu (26,900); m, sample quality (g).

Chlorophyll and carotenoid content were determined as follows. Fresh leaf samples (0.5 g) were rapidly ground to homogeneity in 2–3 mL 95% ethanol. Then, 10 mL 95% ethanol was added and the sample was further ground to a pulp before being left aside for 3–5 min. The extract was filtered into a brown volumetric bottle, and diluted to 15 mL with 95% ethanol. Absorbance values were measured at 665, 649, and 470 nm, with 95% ethanol used as a blank. Three independent technical replicates were conducted for each experiment. The following equations were used to determine chlorophyll and carotenoid contents:

$$ {\displaystyle \begin{array}{c}\mathrm{C} chl=\mathrm{C}a+\mathrm{C}b\\ {}\mathrm{C}\mathrm{hlorophyll}\;a\;\mathrm{content}\ \left(\mathrm{mg}\cdotp {\mathrm{g}}^{-1}\mathrm{FW}\right)=13.95\mathrm{A}665\ \mathrm{nm}-6.88\mathrm{A}649\ \mathrm{nm}\\ {}\mathrm{C}\mathrm{hlorophyll}\ b\;\mathrm{content}\ \left(\mathrm{mg}\cdotp {\mathrm{g}}^{-1}\mathrm{FW}\right)=24.96\mathrm{A}649\ \mathrm{nm}-7.32\mathrm{A}665\ \mathrm{nm}\\ {}\mathrm{Total}\ \mathrm{chlorophyll}\ \mathrm{content}\ \left(\mathrm{mg}\cdotp {\mathrm{g}}^{-1}\mathrm{FW}\right)=\mathrm{C}\ \left(\mathrm{mg}\cdotp {\mathrm{g}}^{-1}\right)=\left(\mathrm{C} chl\times \mathrm{V}\times \mathrm{n}\right)/\mathrm{m}\\ {}\mathrm{C}\mathrm{arotenoid}\ \mathrm{content}\ \left(\mathrm{mg}\cdotp {\mathrm{g}}^{-1}\mathrm{FW}\right)=\mathrm{C}\ \left(\mathrm{mg}\cdotp {\mathrm{g}}^{-1}\right)=\frac{\left(1000\times {A}_{470}-2.05\times {C}_a-114.8\times {C}_b\right)\times V\times n}{245\times m}\end{array}} $$

C_*a*_, chlorophyll a; C_*b*_, chlorophyll b; V, total volume of extract (mL); n, dilution time; m, sample weight (g).

### Statistical analyses

The results represent the means±SE of three replicates. Statistical analyses were performed using the software IBM SPSS Statistics 23.0 (https://www.ibm.com/docs/en/spss-statistics/23.0.0). The data of qRT–PCR and pigment content analysis were compared by T–test and ANOVA (Tukey test), respectively. A *p* value less than 0.05 was considered statistically significant.

## Supplementary Information

**Additional file 1: Table S1.** Transcription factors (TFs) in C2 versus C3. **Table S2.** Transcription factors (TFs) in C1 versus C2.

**Additional file 2: Table S3.** List of gene*–*specific primers used for qRT–PCR test.

## Data Availability

The datasets generated and/ or analysed during the current study were mostly included in this article. The reference genomes of *B. oleracea* are available from EnsemblPlants genome database (http://plants.ensembl.org/Brassica_oleracea/Info/Index) and Bolbase (http://ocri-genomics.org/bolbase/). The plant materials and the raw RNA–seq data during the current study are available from the corresponding author on reasonable requests. The datasets supporting the conclusions of this article are included within the article (and its additional file).

## Abbreviations

*DFR*: *Dihydroflavonol–4 reductase*
*HCT*: *Shikimate O–hydroxycinnamoyltransferase*
*PPD*: *Pheophorbidase*
*crtB*: *15–cis–phytoene synthase*
*PAL*: *Phenylalanine ammonia–lyase*
*CHS*: *Chalcone synthase*
*ANS*: *Anthocyanin synthase*
*MYB*: *Myeloblastosis*
*PIF3*: *Phytochrome interacting factor 3*
*HY5*: *Elongated hypocptyl 5*
*HemA*: *Glutamyl*–*tRNA reductase*
*FLU*: *Fluorescent* in *blue light*
MEP: 2–C–methyl–D–erythritol–4–phosphate
MVA: Mevalonate
*PSY*: *Phytoene synthase*
*CRTISO*: *Carotenoid isomerase*
ε–*LCY*: *Epsilon lycopene cyclase*
*LCYE*: *Lycopene epsilon cyclase*
*CCD*: *Carotenoid cleavage dioxygenase*
*ZDS*: *Carotene desaturase*
bHLH: Basic helix–loop–helix protein
WD40: WD40 repeat–containing protein
*TTG1*: *Transparent testa glabra1*
*TT8*: *Transparent testa 8*
*MYBL2*: *MYB*–*like 2*
*TT19*: *Transparent testa 19*
*C4H*: *Cinnamate–4–hydroxylase*
*TIR1*: *Transport inhibitor response 1*
*LBD39*: *Lateral organ boundaries domain 39*
*NAC*: *No apical meristem*
WRKY: WRKY DNA–binding protein
*UGT*: *UDP–glucuronosyltransferase*
*GST*: *Glutathione S–transferase*
*CRD*: *Beta 1*, *3 glucan synthase catalytic subunit*
*PORC*: *Protochlorophyllide oxidoreductase C*
*CAO*: *Chlorophyll a oxygenase*
*CLH*: *Chlorophyllase*
*PAL*: *Phe–ammonia lyase*
*4CL*: *4–coumarate:CoA ligase*
*CHI*: *Chalcone isomerase*
*F3’H*: *Flavonoid 30–hydroxylase*
*F3H*: *Flavanone 3–hydroxylase*
*CPOX*: *Coproporphyrinogen–III oxidase*
*ChlH*: *A subunit of Mg–chelatase*
*NOL*: *Chlorophyll b reducatase*
*RCCR*: *Red chlorophyll catabolite reductase*
*HemN*: *Oxygen–independent coproporphyrinogen III oxidase*
*chlM*: *Magnesium–protoporphyrin O–methyltransferase*
*chlE*: *Magnesium–protoporphyrin IX monomethyl ester (oxidative) cyclase*
*por*: *Protochlorophyllide reductase*
*HCAR*: *7–hydroxymethyl chlorophyll a reductase*
*PAO*: *Pheophorbide a oxygenase*
*HemD*: *Uroporphyrinogen–III synthase*
*HemE*: *Uroporphyrinogen decarboxylase*
*HemY*: *Protoporphyrinogen/coproporphyrinogen III oxidase*
*DVR*: *Divinyl chlorophyllide a 8–vinyl–reductase*
*chlG*: *Chlorophyll a synthase*
*ZEP*: *Zeaxanthin epoxidase*
*VDE*: *Violaxanthin de–epoxidase*
*NCED*: *9–cis–epoxycarotenoid dioxygenase*
*PDS*: *15–cis–phytoene desaturase*
*ZDS*: *Zeta–carotene desaturase*
*lcyB*: *Lycopene beta–cyclase*
*crtZ*: *Beta–carotene 3–hydroxylase*
*crtH*: *Prolycopene isomerase*
